# Family member incarceration and coping strategies during the COVID-19 pandemic

**DOI:** 10.1186/s40352-021-00142-w

**Published:** 2021-07-09

**Authors:** Alexander Testa, Chantal Fahmy

**Affiliations:** Department of Criminology & Criminal Justice, The University of Texas at San Antonio, 501 W. Cesar Chavez Blvd., San Antonio, TX 78207 USA

**Keywords:** Incarceration, COVID-19, Coping, Health, Family member

## Abstract

**Background:**

The disproportionately high rate of incarceration and COVID-19 cases during the summer of 2020 in the United States contributed to a set of circumstances that has produced considerable public health concerns as correctional facilities have emerged as significant COVID-19 hot spots. During the COVID-19 pandemic, having a family member incarcerated can be an especially stressful experience. This study assesses how concern about an incarcerated family member contracting COVID-19 impacts diverse coping strategies.

**Results:**

Data are from a survey of individuals who have a family member incarcerated in Texas (*N* = 365). Ordinary least squares regression is used to examine the association between concern about an incarcerated family member contracting COVID-19 and coping strategies. Findings demonstrate that higher levels of concern for an incarcerated person’s wellbeing during the COVID-19 pandemic is associated with dysfunctional coping mechanisms, but not adaptive or functional coping strategies.

**Conclusions:**

Results suggest appropriate systemic responses by correctional administrations and public health practices can help mitigate dysfunctional coping mechanisms by family members during infectious disease outbreaks in correctional facilities.

## Introduction

As of August 2020, the United States had approximately one-quarter of the world’s prisoners and COVID-19 cases, despite having just 5% of the global population (Johns Hopkins University, 2020; Walmsley, 2018); a combination ripe for adverse public health outcomes. At present, the United States has the highest incarceration rate in the world (Walmsley, 2018), and correctional facilities are characterized by disparate exposure to infectious diseases via overcrowded living spaces, poor ventilation, shared hygiene facilities, reuse of contaminated drug needles, and more (Akiyama et al., 2020; Massoglia & Pridemore, 2015). Together, these conditions have produced a catastrophic situation with correctional facilities emerging as key COVID hot spots (Burki, 2020).

Many people behind bars are plagued with health issues prior to entering prison, with incarceration serving as an accelerator that exacerbates poor health outcomes (Akiyama et al., 2020; Golembeski & Fullilove, 2005). Considering the rapid spread of infectious diseases through prisons and jails (Beaudry et al., 2020), and emerging evidence on the role of cycling through correctional facilities for increased community transmission (Reinhart & Chen, 2020, 2021), the spread of COVID-19 undoubtedly serves as a stressful event both for incarcerated populations and their family and friends on the outside. A recent body of research documents the health-related consequences—particularly for women—of having a family member incarcerated (Wildeman et al., 2019). Health-related consequences are essentially unintended effects within the domain of physical health and emotional well-being experienced by family members on the outside who have a loved one incarcerated and may range from depression to substance abuse to cardiovascular disease (Bruns & Lee, 2020; Comfort, 2007; Wildeman et al., 2019). For instance, Lee et al. (2014) found an increased likelihood of self-reported medical diagnoses of poor health across conditions such as obesity, diabetes, and heart attack or stroke for women, but not men, with a family member incarcerated. During the COVID-19 pandemic, family members of the incarcerated are afflicted with their own uneasiness linked to the pandemic alongside the anguish of having an incarcerated family member. For example, during data collection for the current study, a woman whose husband was incarcerated during the pandemic, explained to us that lack of information about his COVID-positive test has caused her “a great deal of anxiety” which has led to a “severe panic attack and extreme depression”.

Diverse psychosocial responses by loved ones on the outside can inflict acute and long-term mental health consequences (Gurvich et al., 2020). In order to reduce the effects of stressors, such as the COVID-19 pandemic, individuals tend to utilize a range of coping strategies. Functional or adaptive coping strategies have the potential to effectively mitigate the psychological responses to stress whereas dysfunctional coping strategies tend to heighten the impact and exacerbate any prior underlying health conditions (Gurvich et al., 2020; Kar et al., 2020). For example, blaming one’s self—a form of dysfunctional coping—for a loved one’s incarceration does little to alleviate the stress felt. Contrarily, positively reframing the incarceration as a time for their loved one to get clean from substance use, for instance, may prove to be a more adaptive coping strategy that reduces the taxing impact on health. Add to this the uncertainty of COVID-19’s influence on incarcerated populations and it is clear why their loved ones are attempting to decrease the effects of mental distress. In fact, since the COVID-19 outbreak began, the differential effects of various coping strategies on depression, anxiety, stress, and suicidality can be seen globally, are more pronounced for women, and may persist long after the routines of life return (Chew et al., 2020; Gurvich et al., 2020).

The current study extends prior research on the various coping styles of people with an incarcerated loved one during the COVID-19 pandemic. Specifically, we draw on data collected from a non-profit organization in Texas that serves those with a loved one incarcerated (*n =* 365; respondents are 93.7% female) to examine a variety of functional and dysfunctional coping strategies among individuals who are concerned about their loved one contracting the virus while incarcerated.

### Data

Data are from a cross-sectional survey of individuals who have a family member incarcerated. Respondents are members of a non-profit organization tailored to persons who have family members incarcerated in Texas —Texas Inmate Family Association (TIFA). Surveys were disseminated through Qualtrics between July and August 2020. Participation in the study was voluntary and those who participated in the survey were enrolled in a chance to get receive a $15 electronic Walmart gift card after informed consent was granted. In total, 517 respondents participated in the survey, although several respondents opted to not answer all questions. The current study uses data from 365 individuals with valid responses to all relevant questions. Patterns of missing data are reported in Table 4, Appendix 1. The study was approved by ] The University of Texas at San Antonio Institutional Review Board.

### Dependent variable

**Fig. 1:**
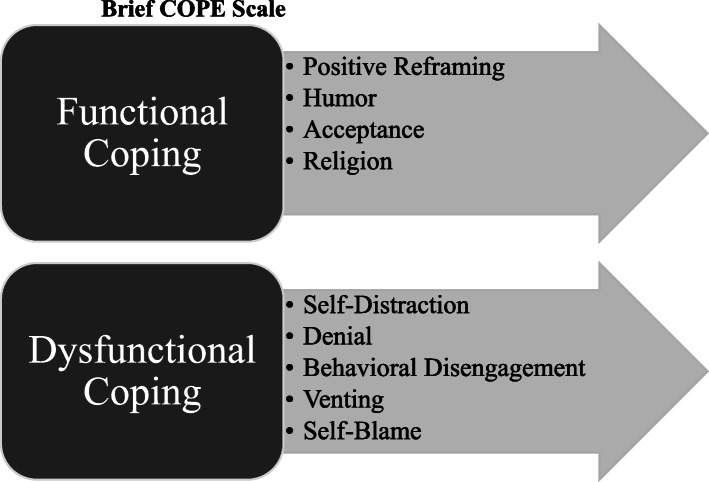
Brief COPE Scale

The dependent variables measure dysfunctional and functional coping strategies derived from the Brief COPE inventory (Carver, 1997). The Brief COPE is an abbreviated version of the original 60-item COPE inventory (Carver et al., 1989), which measures how people effectively or ineffectively manage distress in response to stressors, particularly in health-related research. The Brief COPE has demonstrated reliability and validity in numerous contexts, in different languages, and among diverse stressors (see e.g., Cooper et al., 2006; Kapsou et al., 2010; Muniandy et al., 2021). The scale can be classified into functional or dysfunctional clusters (Coolidge et al., 2000). Functional or adaptive coping involves cognitive strategies used to directly reduce the emotional anguish caused by the stressful situation whereas dysfunctional coping is considered a maladaptive strategy that does not alleviate the impact of stressors (Carver, 1997). The core themes of the Brief COPE scale are presented visually in Fig. 1. Respondents were asked 18 questions related to how they cope with their loved one’s incarceration. These items are listed in Table 1. Specifically, respondents were asked how often they engage in said behavior ranging from “never” (0), “a little bit” (1), “a medium amount” (2), or “a lot” (3) when thinking about the person’s incarceration. Responses were summed into additive scales ranging from 0 to 24 for dysfunctional coping (Cronbach’s alpha = .749) and 0 to 21 for functional coping (Cronbach’s alpha = .675).

**Table 1:** Brief COPE Measures

***Dysfunctional Coping Measures***	
*Please indicate whether you “never” do this, you do this “a little bit”, you do this “a medium amount”, or you do this “a lot” when thinking about the person’s incarceration.*	
Self-Distraction 1	I’ve been turning to work or other activities to take my mind off things
Self-Distraction 2	I’ve been doing something to think about it less, such as watching television, reading, daydreaming, sleeping, or exercising
Denial 1	I’ve been saying to myself “this isn’t real
Denial 2	I’ve been refusing to believe that it has happened
Behavioral Disengagement 1	I’ve been giving up trying to deal with it
Behavioral Disengagement 2	I’ve been giving up the attempt to cope
Venting 1	I’ve been saying things to let my unpleasant feelings escape
Venting 2	I’ve been expressing my negative feelings
Self-Blame 1	I’ve been criticizing myself
Self-Blame 2	I’ve been blaming myself for things that happened
***Functional Coping Measures***	
*Please indicate whether you “never” do this, you do this “a little bit”, you do this “a medium amount”, or you do this “a lot” when thinking about the person’s incarceration.*	
Positive Reframing 1	I’ve been trying to see it in a different light, to make it seem more positive
Positive Reframing 2	I’ve been looking for something good in what is happening
Humor 1	I’ve been making jokes about it
Humor 2	I’ve been making fun of the situation
Acceptance 1	I’ve been accepting the reality of the fact that it has happened
Acceptance 2	I’ve been learning to live with it
Religion 1	I’ve been trying to find comfort in my religion or spiritual beliefs
Religion 2	I’ve been praying or meditating

### Independent variable

COVID-19 concern is measured using a question asking the respondent “Since the COVID-19 pandemic began (March 2020), how concerned are you about the incarcerated person contracting COVID-19?” Responses included “not at all concerned”, “a little concerned”, “somewhat concerned”, and “very concerned”. Since only 5 respondents stated they were “not concerned at all” this response was combined with “a little concerned”.

### Control variables

Control variables include respondent race/ethnicity (White [reference], Black, Hispanic, other race/ethnicity), respondent age categories (younger adult: 40 or younger, middle aged adult: 41–59 years old, and older adult: 60 or older), respondent sex (1 = female, 0 = male), whether the respondent is currently married (1 = currently married; 0 = not married), respondent’s education level (1 = college graduate; 0 = less than college), and self-rated health—"In general, would you say your physical health is excellent, good, fair, or poor?"—(1 = good/excellent; 0 = poor/fair). A scale of material hardship asks respondents whether in the prior 12 months there was a time when they or their household experienced the following: (1) evicted from their home/apartment, (2) had phone services disconnected, (3) worried food would run out, (4) could not pay the full amount of utility bills, (5) could not pay rent or mortgage, and (6) had utility services cut off (Cronbach’s alpha = .807). Measures regarding the incarcerated person include a binary measure of whether the focal person had previously been incarcerated (1 = yes, 0 = no), the respondent’s relationship to the incarcerated person (child, spouse, or other),^1^ and the type of crime the individual was convicted of (violent offense, sex offense, drug/alcohol offense, or other offense).

## Method

We begin by displaying the descriptive statistics of the analytic sample. Next, we assess association between COVID concern and functional (i.e., adaptive) and dysfunctional (i.e., maladaptive) coping strategies using ordinary least squares (OLS) regression. Supplemental analyses assess variation in COVID concern across individual coping items.

## Results

Table 2 presents the summary statistics. Approximately 5.8% of the sample reported having none or little COVID concern, 15.6% reported being somewhat concerned, and 78.6% reported being very concerned. Across demographic characteristics, most of the sample are 60 or older (51.0%) followed by 41–59 years old (35.6%), with the smallest category being 40 and younger (13.4%). The sample is largely composed of females (93.7%), and most respondents are White (72.3%) with fewer Hispanic (17.0%) and Black (9.6%) respondents.

**Table 2:** Summary Statistics of Analytic Sample (*N* = 365)

Variables	Mean	Standard Deviation	Minimum	Maximum
*Dependent Variable*				
Dysfunctional Coping	10.25	5.26	0	24
Functional Coping	13.16	4.02	0	21
*Independent Variable*				
COVID Concern: None or Little	5.8%	–	0	1
COVID Concern: Somewhat	15.6%	–	0	1
COVID Concern: Very	78.6%	–	0	1
*Age Categories-*				
40 or younger	13.4%	–	0	1
41–59 years old	35.6%	–	0	1
60 or older	51.0%	–	0	1
Female	93.7%	–	0	1
*Respondent Race/Ethnicity*				
White	72.3%	–	0	1
Black	9.6%	–	0	1
Hispanic	17.0%	–	0	1
Other	1.1%	–	0	1
Married	60.8%	–	0	1
College Graduate	37.5%	–	0	1
Good Health	64.9%	–	0	1
Hardship Scale	0.60	1.23	0	6
*Relationship to Incarcerated Person*				
Child	52.9%	–	0	1
Spouse	33.4%	–	0	1
Other	13.7%	–	0	1
Prior Incarceration	36.2%	–	0	1
*Crime Type of Incarcerated Person*				
Violent	51.0%	–	0	1
Sex Offense	21.9%	–	0	1
Drug/Alcohol	18.9%	–	0	1
Other	8.2%	–	0	1

**Fig. 2:**
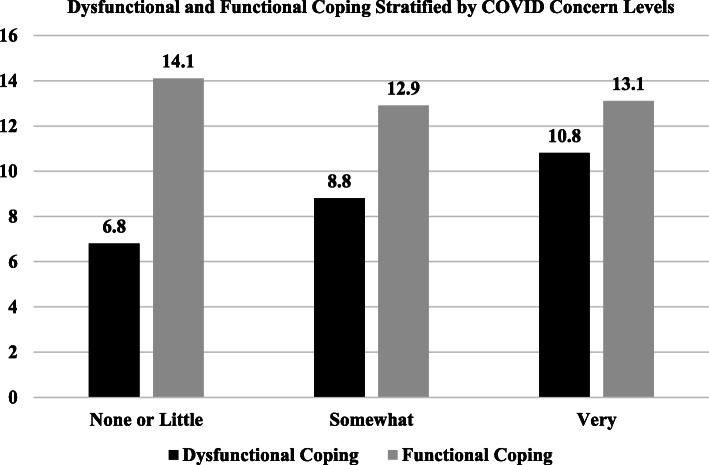
Dysfunctional and Functional Coping Stratified by COVID Concern Levels

Figure 2 shows the mean levels of dysfunctional and functional coping items stratified by COVID concern levels. Results show that dysfunctional coping is lowest among those with none/little COVID concern (6.8), but higher among respondents who report being somewhat (8.8) or very (10.8) concerned about the incarcerated person contracting COVID-19. However, levels of functional coping remained approximately similar across all levels of COVID concern.

Table 3 examines these results in a multivariate context. Findings show that net of control variables, respondents who reported being somewhat (*b* = 2.023, *p* < .05) or very (*b* = 3.823, *p* < .001) concerned about the incarcerated person contracting COVID-19 had a positive association with dysfunctional coping, relative to respondents who reported none or little concern. Findings pertaining to functional coping demonstrated no association between COVID concern and functional coping.^2^ Supplemental analyses investigating the association between COVID concern and each individual item in the coping scale are presented in Table 5, in Appendix 2 (dysfunctional coping) and Table 6, Appendix 3 (functional coping). These findings show that COVID concern was unrelated to any of the individual functional coping strategies. However, COVID concern was related to each of the dysfunctional coping strategies, apart from self-distraction 1 and both venting items.

**Table 3:** OLS Regression of Coping Strategy on COVID Concern (*N* = 365)

	Dysfunctional Coping		Functional Coping	
COVID Concern	***b***	95% CI	***b***	95% CI
None or Little (Reference)	–	–	*–*	–
Somewhat	2.023*	(.020, 4.026)	−1.247	(−3.525, 1.031)
Very	3.823***	(2.128, 5.517)	−.979	(−3.097, 1.140)

Control variables include respondent sex, respondent race/ethnicity, marital status, college graduate, good health, hardship scale, relationship to incarcerated person, prior incarceration, and crime type.

***p < .001, *p < .05

## Discussion

Building on previous work, the current study integrated two areas of research: the literature on coping and literature on the collateral consequences of incarceration for families. In doing so, we have expanded work in these areas to further elucidate the effects of the COVID-19 pandemic on individuals with a loved one incarcerated during the pandemic in Texas. Nearly 88% of the sample noted they were somewhat concerned or very concerned about their loved one contracting COVID-19 while in prison. Their concern is valid since the COVID-19 rate is roughly six times higher for prison populations than that of the general U.S. population (Saloner et al., 2020). We found that people who were most concerned about their loved one contracting COVID-19, exhibited many coping strategies that were highly dysfunctional by nature. For example, blaming one’s self, behavioral disengagement, or being in a state of denial are not active ways of engaging with the stressor and may aggravate mental health symptoms. Conversely, we found that regardless of level of concern (i.e., not concerned to very concerned) regarding their loved one contracting COVID-19, functional coping strategies by and large were not utilized to alleviate stress. In other words, respondents neglected to positively reframe, use religion or humor, or accept the current situation related to the possibility of their family member contracting the virus; thereby disregarding coping strategies focused on the root of the problem. Importantly, our supplemental analyses showed that COVID concern was related to each category of dysfunctional coping except for venting (see Table 5 in Appendix 2). While the exact mechanisms behind this are not immediately clear, it may be that those with a family member incarcerated are more socially isolated —especially during COVID-19— and thus less likely to have support networks as an outlet or to utilize externalized dysfunctional coping strategies, such as venting. On the other hand, the social isolation may lead them to be more likely to use internalized coping such as self-distraction, denial, behavioral disengagement, and self-blame.

While the present study presents a novel look at the impacts of the COVID-19 pandemic on those with a loved one incarcerated, there are limitations that future research can expand upon. First, results are based on a small, local sample in Texas and may not be generalizable to other populations. Second, at the time the survey was conducted (summer of 2020), the pandemic’s grip reached a peak in the United States and the potential for widespread vaccine distribution had not yet been formulated. Perhaps level of concern on the part of the respondent was heightened during this time. Future research should investigate how the degree of concern and coping styles varied throughout the duration of the pandemic. Third, although we aimed to identify the overarching coping styles used by respondents, we did not examine how functional or dysfunctional coping may prompt other health-related issues in this population. Future research should build upon our work and explore how various coping styles can lead to beneficial or deleterious health repercussions.

Despite these limitations, our research demonstrates the value in understanding the ever-changing dynamics presented by the pandemic and mass incarceration in terms of practical implications. Clearly, there is a need for the population as a whole to focus on more functional coping strategies when dealing with stress, but particularly for those with an incarcerated loved one whose health status is unknown. Nonprofit organizations such as the Texas Inmate Family Association (TIFA) are positioned in a way to help increase functional coping strategies and supportive practices to alleviate negative coping mechanisms. Additionally, correctional administrations have myriad opportunities to revise policies in light of the pandemic (Novisky et al., 2020), especially since a majority of the COVID-19 clustered outbreaks have occurred in prisons and jails (The New York Times, 2020). At the very least, corrections departments, such as the Texas Department of Criminal Justice (TDCJ), should facilitate enhanced contact between the incarcerated person and their loved ones on the outside. With face-to-face visitation suspended across jurisdictions, the use of remote video visitation is critical. Access to means which allows maintenance of contact and social ties is of utmost importance, particularly because reduced abilities to verbalize with loved ones “may raise anxieties and fears about risks for infection, both personally and out of concern for loved ones” (Novisky et al., 2020, p. 1246). Simply, the ability to regularly talk to their loved one may relieve family and friends' concerns.

More broadly, the findings here speak to the ways that social and emotional support mechanisms can potentially be helpful to those with a loved one incarcerated. To be sure, prior research has found social and emotional support to be a key mechanism that can buffer the harmful impacts of incarceration and improve the well-being of both formerly incarcerated individuals (Fahmy, 2021; Fahmy & Wallace, 2019; Wallace et al., 2016) and those with a family member incarcerated (Testa & Fahmy, 2021). Related to COVID-19, research has also documented that among the general population, social support can buffer the link between worry about COVID-19 and psychological well-being (Grey et al., 2020; Szkody et al., 2020). Correctional facilities can help foster greater social and emotional support by connecting those with a family member incarcerated to outside support agencies such as TIFA, as well as other local and national organizations that support children and families of the incarcerated. For instance, the Children’s Bureau of the U.S. Department of Health & Human Service maintains a list of such organizations that can be disseminated to prisons and jails nationwide (Child Welfare Information Gateway, 2021). Down the road, as the pandemic breaks it may also be especially pressing for correctional facilities to reopen visitation opportunities and do so in a manner that enables those who share the experience of having a loved one incarcerated to interact as a means of fostering greater interpersonal support. For instance, Arditti (2005, p. 258) has previously suggested “friendlier visiting areas that provide activities for children while they wait may also free visitors up to interact with each other and provide informal social and emotional support.”

Finally, the toll of the COVID-19 pandemic on incarcerated persons and their family members also points to decarceration (i.e., reducing the number of people incarcerated) as a policy option that warrants greater consideration. Indeed, incarceration is harmful to the health of both communities and families (Gifford, 2019; Wildeman et al., 2019), and these harms may be exacerbated during the COVID-19 pandemic (Novisky et al., 2021; Reinhart & Chen, 2020, 2021. Evidence suggests that a release of those who do not pose an ongoing threat of danger can help flatten the curve of COVID-19 with minimal risk to public safety (Malloy et al., 2021; Vest et al., 2021) and such a policy was a recommended component of the National Academies’ October 2020 expert policy consensus (National Academies of Sciences, Engineering, and Medicine, 2021). The results of the current study also suggest such an approach may minimize apprehension stemming from loved ones and simultaneously reform criminal justice policies that have contributed to mass incarceration (Macmadu et al., 2020; Novisky et al., 2020).

## Data Availability

The datasets generated and/or analyzed during the current study are not publicly available due to confidentiality restrictions.

## Appendix 1

**Table 4:** Patterns of Missing Data

Selection criteria:	
Participated in the survey	(*n* = 517)
	**↓**
Has data on respondent age	(*n* = 467)
	**↓**
Has data on coping	(*n* = 431)
	**↓**
Has data on control variables	(*n* = 365)

## Appendix 2

**Table 5:** OLS Regression of COVID Concern on Individual Dysfunctional Coping Items (*N* = 365)

Dysfunctional Coping Items		
COVID Concern	***b***	95% CI
**Self-Distraction 1**		
Somewhat	.196	(−.324, .717)
Very	.406	(−.073, .884)
**Self-Distraction 2**		
Somewhat	.223	(−.277, .723)
Very	.487*	(.031, .943)
**Denial 1**		
Somewhat	.284	(−.211, .778)
Very	.627**	(.188, 1.066)
**Denial 2**		
Somewhat	.252	(−.041, .545)
Very	.382***	(.161, .603)
**Behavioral Disengagement 1**		
Somewhat	.380*	(.046, .714)
Very	.165	(−.082, .413)
**Behavioral Disengagement 2**		
Somewhat	.270**	(.098, .442)
Very	.376***	(.261, .490)
**Venting 1**		
Somewhat	−.067	(−.435, .301)
Very	.136	(−.189, .461)
**Venting 2**		
Somewhat	.081	(−.247, .408)
Very	.132	(−.149, .413)
**Self-Blame 1**		
Somewhat	.201	(−.150, .552)
Very	.612***	(.299, .926)
**Self-Blame 2**		
Somewhat	.203	(−.095, .501)
Very	.501***	(.245, .756)

Control variables include respondent sex, respondent race/ethnicity, marital status, college graduate, good health, hardship scale, relationship to incarcerated person, prior incarceration, and crime type.

****p* < .001, ***p* < .01, **p* < .05

## Appendix 3

**Table 6:** OLS Regression of COVID Concern on Individual Functional Coping Items (*N* = 365)

Functional Coping Items		
COVID Concern	***b***	95% CI
**Positive Reframing 1**		
Somewhat	−.339	(−.841, .163)
Very	−.292	(−.751, .168)
**Positive Reframing 2**		
Somewhat	−.012	(−.590, .567)
Very	.055	(−.483, .593)
**Humor 1**		
Somewhat	.077	(−.208, .363)
Very	.030	(−.218, .277)
**Humor 2**		
Somewhat	.061	(−.114, .235)
Very	−.000	(−.142, .141)
**Acceptance 1**		
Somewhat	−.139	(−.553, .275)
Very	−.062	(−.433, .310)
**Acceptance 2**		
Somewhat	−.384	(−.831, .062)
Very	−.242	(−.654, .170)
**Religion 1**		
Somewhat	−.228	(−.695, .239)
Very	−.213	(−.640, .215)
**Religion 2**		
Somewhat	−.282	(−.647, .082)
Very	−.255	(−.578, .067)

Control variables include respondent sex, respondent race/ethnicity, marital status, college graduate, good health, hardship scale, relationship to incarcerated person, prior incarceration, and crime type.

## Footnotes

1 The “other” category captures several categories with samples too small to independently analyze including sibling (*n* = 12), extended family member (i.e., cousin or uncle; *n* = 8), friend (*n* = 15), and grandchild (*n* = 8).

2 Considering that the sample is mostly female, we conducted a sensitivity analysis removing males from the sample. The results of that analysis provided substantively similar findings to the models reported using the full sample. Because of the small number of males in the sample (*n* = 23), analysis of the subsample of males generated a large degree of error and unreliable estimates. These analyses are available upon request.
